# Kidney function is associated with severity of white matter hyperintensity in patients with acute ischemic stroke/TIA

**DOI:** 10.1186/s12883-016-0714-0

**Published:** 2016-10-06

**Authors:** Lixia Zong, Ming Yao, Jun Ni, Lixin Zhou, Jing Yuan, Bin Peng, Yi-Cheng Zhu, Liying Cui

**Affiliations:** 1 Department of Neurology, Peking Union Medical College Hospital, Chinese Academy of Medical Sciences, No.1 Shuaifuyuan, Beijing, 100730 China; 2 Department of Neurology, Peking Union Medical College Hospital, Neuroscience Center, Chinese Academy of Medical Sciences and Peking Union Medical College, No.1 Shuaifuyuan, Beijing, 100730 China

**Keywords:** Renal dysfunction, White matter hyperintensity, Chronic kidney disease, Stroke

## Abstract

**Background:**

Previous studies suggested the potential interactions between cerebrovascular diseases and impaired renal function. However, the relationship between renal function and white matter hyperintensity (WMH), marker of cerebral small vessel disease, in patients with acute ischemic stroke (AIS) or transient ischemic attack (TIA) remains unknown.

**Methods:**

We consecutively enrolled 1632 subjects with AIS or TIA who underwent brain MRI for this analysis. The severity of WMH in both of periventricular (PVH) and deep subcortical white matter (SDWMH) was evaluated using Fazekas scale. Estimated glomerular filtration rate (eGFR) was calculated by the equation of the Modification Diet for Renal Disease. Multinomial logistic regression was performed to evaluate the association between the severity of WMH and eGFR.

**Results:**

Advanced age and hypertension were independently associated with the severity of both PVH and SDWMH (all *p* < 0.001). There is a significantly inverse association between eGFR and PVH. Patients having each 30 ml/min/1.73 m^2^ increase in eGFR was associated with 75 % of risk of having degree 3 of WMH in periventricular areas compared with degree 0 (*p* = 0.04, OR = 0.75, 95 % CI 0.61–0.92). However this inverse association was not found between eGFR and SDWMH (*P* = 0.50, OR = 0.93, 95 % CI0.75–1.14).

**Conclusion:**

Our study demonstrates that renal dysfunction (eGFR) is independently associated with the severity of PVH but not SDWMH in patients with acute ischemic stroke. This results highlighted different pathological mechanism and risk factors of PVH and SDWMH.

## Background

Chronic kidney disease has been recognized as a rapidly growing global health burden in the past decade. Previous studies showed that individuals with an eGFR below 60 mL/min per 1.73 m^2^ had a higher risk of stroke and vice versa, suggesting the potential interactions between cerebrovascular diseases and impaired renal function [1, 2].

The regulation of the microvasculatures of brain and kidney is functionally similar. In addition, kidney impairment is characterized by glomerular endothelial dysfunction and lipohyalinosis, both of which are features of small-artery diseases [1, 3]. Thus people can readily presume that there might be association between kidney function and silent MRI changes which related to cerebral small vessel disease, like white matter hyperintensity (WMH), lacune and microbleeds. Previous studies showing independent associations between chronic kidney disease (CKD) and WMH in individuals without stroke supported the above mentioned hypothesis [4–6]. However, data on stroke patients have been subjected to debate [7, 8]. WMH burden was related to increased risk of stroke and unfavorable post-stroke outcomes, so was impaired renal function [9–11]. Therefore the relationship between WMH and renal failure in ischemic stroke patients still needs more investigations. Moreover, although WMH in periventricular area and in deep white matter were regarded to have different pathological features [12], studies evaluated the lesions respectively in two locations were rare.

In the present study based on data collected in a large cohort of Chinese patients with acute ischemic stroke (AIS) or transient ischemic attack (TIA), we aimed to explore the potential risk factors of WMH, particularly the association between renal function and WMH.

## Methods

### Patients

Data were obtained from the SMART study, a multicenter trial designed to assess the effectiveness of a guideline-based structured care program for secondary stroke prevention in China. The complete study protocol, approved by the ethics committees at Peking Union Medical College Hospital, has been detailed elsewhere [13] and the reference number is S-151. Informed written consent was obtained from each patient.

Briefly, between April 2008 and December 2010, a total of 3821 patients, aged > 18 years, with cerebral ischemic infarct or TIA within 30 days were enrolled. Patients who had severe comorbidities including heart failure, respiratory failure, renal failure, severe liver dysfunction, malignancy were excluded. For this study, only the 1975 patients who had brain MRI examination were included. 233 scans were excluded because of motion artifacts, leaving 1752 patients for WMH evaluation. Among patients who had WMH evaluation, those who had missing data on cardiovascular risk factors were further excluded so that the final sample was composed of 1632 subjects.

### Definition of risk factors

A history of ischemic heart disease (IHD) was considered if a history of myocardial infarction, bypass cardiac surgery, or angioplasty was recorded. Atrial fibrillation (AF) was considered according to the electrocardiogram manifestation on admission or previously documented diagnosis. A history of ischemic stroke/TIA was considered according to previously documented diagnosis.

Diabetes mellitus (DM) was considered present when fasting blood glucose level ≥ 7.0 mmol/L, or antidiabetic drugs were taken or a current history of DM was reported. Hypertension was defined by high blood pressure (systolic blood pressure ≥ 140 mmHg or diastolic blood pressure ≥ 90 mmHg), or by use of antihypertensive drugs, or previous diagnosis. Hypercholesterolemia was defined as total cholesterol ≥ 5.2 mmol/L or lipid-lowing drugs were taken or a current history was documented. Smoking habits were categorized as nonsmokers and smokers (former or current). Admission serum creatinine was abstracted from the medical chart. Estimated glomerular filtration rate (eGFR) was calculated using the equation of the Modification Diet for Renal Disease modified by the Chinese coefficient [14]: eGFR(ml/min/1.73 m^2^) = 186 × serum creatinine (exp[−1.154]) × age (exp[−0.203]) × 1.233 × 0.742 (if female). According to the eGFR, CKD stages were defined as follow: stage1:eGFR ≥ 90 ml/min per 1.73 m^2^, stage 2:60 ≤ eGFR < 90 ml/min per 1.73 m^2^, stage3: 30 ≤ eGFR < 60 ml/min per 1.73 m^2^, stage4: 15 ≤ eGFR < 30 ml/min per 1.73 m^2^, stage5: eGFR < 15 ml/min per 1.73 m^2^ [15].

### Rating of WMH

The brain MRI scan had been performed on a 1.5-Tesla or 3.0-Tesla System due to different research centers, then MRI scans were converted from DICOM to analysis format by eZdicom software, and T2 fluid-attenuated inversion recovery (FLAIR) sequences were used to evaluate the degree of WMH. The Fazekas scale [16] was used to score both the severity of periventricular WMH (PVH) and deep subcortical WMH (SDWMH). All images were analyzed by the same experienced reader (X.-F.L.) blinded to all clinical data. The intra-rater agreement for the rating of WMH was assessed on a random sample of 54 subjects at 8-week intervals. The intrarater reliability analysis showed a good reliability with κ values of 0.73 and 0.79 for PVH and SDWMH respectively.

### Statistical analysis

The SPSS Version 19.0 was used for all analyses. The descriptive statistics on the baseline characteristics are presented as well as their crude distribution according to WMH degrees. The continuous variables were summarized as mean ± SD or median with interquartiles, and all the categorical variables were presented as number (percent). Univariate analysis was used to evaluate the relationship between WMH and other variables (data not shown), and a nominal *P* value less than 0.2 was used to select variables to build the multinomial regression model. For multinomial logistic regression, with both PVH and SDWMH rated with a 4-degree score as the dependent variable, each response category was contrasted against the reference category (degree 0). Each model adjusted on age, gender and hypertension at least. Statistical significance level for all analyses was set at *P* value less than 0.05.

## Results

Baseline characteristics of the study sample are shown in Table 1. The mean age was 62.3 years (SD = 11.5) and 1118 (68.5 %) subjects were male. As the patients with severe comorbid illness had been excluded on admission, there are few people with CKD of stage 4 and 5 (5 for stage 4 and none for stage 5), and only 59 (3.6 %) patients had an eGFR below 60 ml/min per 1.73 m^2^ (Total of CKD stage 3, 4 and 5).

**Table 1:** Baseline characteristics of the participants

Characteristic	Total
	N (%)
Age, years*	62.3 ± 11.5
Male gender	1118 (68.5 %)
Smoking	776 (47.6 %)
Diabetes mellitus	508 (31.1 %)
Hypertension	1372 (84.1 %)
Hypercholesterolemia	723 (44.3 %)
Serum creatinine, umol/L	77.4 (65.9–90.0)
eGFR, ml/min/1.73 m^2^	106.9 (89.3–127.9)
Atrial fibrillation	69 (4.2 %)
Ischemic heart disease	187 (11.5 %)
History of ischemic stroke/TIA	434 (26.6 %)
CKD stages	
Stage 1	1211 (74.2 %)
Stage 2	362 (22.2 %)
Stage 3	54 (3.3 %)
Stage 4	5 (0.3 %)
Stage 5	0 (0 %)
PVH degrees	
Degree 0	293 (18.0 %)
Degree 1	749 (45.9 %)
Degree 2	416 (25.5 %)
Degree 3	174 (10.7 %)
SDWMH degrees	
Degree 0	811 (49.7 %)
Degree 1	575 (35.2 %)
Degree 2	131 (8.0 %)
Degree 3	115 (7.0 %)

Variables are presented as mean ± SD (*), median (first-third quartile) or number (percentage)

*Abbreviations*: *eGFR* estimated glomerular filtration rate, *CKD* chronic kidney disease, *PVH* periventricular white matter hyperintensity, *SDWMH* subcortical deep white matter hyperintensity

Of 1632 participants, 811(49.7 %) had no WMH (degree 0) in the subcortical area, 575 (35.2 %) had SDWMH of degree 1, leaving only 246 (15 %) with SDWMH of degree 2 or 3. By contrast, only 293 (18.0 %) participants had no visible WMH in the periventricular area, 749 (45.9 %) participants had PVH of degree 1, 590 (36.2 %) had PVH of degree 2 or 3 (Table 1).

### Renal function and other factors associated with the severity of WMH

The baseline distribution of potential risk factors in relation to WMH degrees and their associations with PVH as well as SDWMH are respectively shown in Tables 2 and 3. Mean age increased with WMH degree in both brain locations (Table 2); each SD increase in age was associated with a higher odds of having higher degrees of WMH. This effect of age is more obvious in PVH than in SDWMH, particularly in degree 3(OR for PVH: 6.98, 95 % CI 5.27–9.25; for SDWMH: 3.45, 95 % CI 2.63–4.53).

**Table 2:** Baseline distribution of potential risk factors across the WMH degrees

	PVH				SDWMH			
	Degree0	Degree 1	Degree 2	Degree 3	Degree0	Degree 1	Degree 2	Degree 3
	*n* = 293	*n* = 749	*n* = 416	*n* = 174	*n* = 811	*n* = 575	*n* = 131	*n* = 115
Age, mean years(SD)	53.6 (10.3)	61.5 (10.4)	65.9 (10.6)	71.7 (8.3)	58.4 (11.0)	64.7 (10.8)	67.7 (9.7)	71.3 (8.8)
Male gender	221 (75.4 %)	490 (65.4 %)	283 (68.0 %)	124 (71.3 %)	584 (72.0 %)	386 (67.1 %)	81 (61.8 %)	67 (58.3 %)
Smoking	170 (58.0 %)	343 (45.8 %)	186 (44.7 %)	77 (44.3 %)	417 (51.4 %)	268 (46.6 %)	53 (40.5 %)	38 (33.0 %)
Diabetes mellitus	77 (26.3 %)	260 (34.7 %)	125 (30.0 %)	46 (26.4 %)	242 (29.8 %)	201 (35.0 %)	33 (25.2 %)	32 (27.8 %)
Hypertention	211 (72.0 %)	635 (84.8 %)	366 (88.0 %)	160 (92.0 %)	640 (78.9 %)	508 (88.3 %)	120 (91.6 %)	104 (90.4 %)
Hypercholesterolemia	131 (44.7 %)	354 (47.3 %)	175 (42.1 %)	63 (36.2 %)	360 (44.4 %)	263 (45.7 %)	62 (47.3 %)	38 (33.0 %)
Serum creatinine, umol/L	75.0 (63.9–85.3)	77.0 (65.0–90.0)	78.1 (66.0–92.8)	84.1 (71.0–96.0)	76.0 (65.0–88.0)	79.0 (66.0–92.0)	79.1 (67.1–92.0)	80.0 (65.0–92.0)
eGFR, ml/min/1.73 m^2^	118.7 (101.9–135.9)	107.3 (89.4–129.5)	102.9 (86.3–123.0)	99.0 (81.0–112.1)	112.2 (93.7–132.3)	102.9 (87.1–123.5)	98.6 (85.4–115.4)	98.7 (83.8–114.8)
Atrial fibrillation	7 (2.4 %)	30 (4.0 %)	21(5.0 %)	11 (6.3 %)	33 (4.1 %)	19 (3.3 %)	6 (4.6 %)	11(9.6 %)
Ischemic heart disease	22 (7.5 %)	72 (9.6 %)	67 (16.1 %)	26 (14.9 %)	72 (8.9 %)	64 (11.1 %)	27 (20.6 %)	24 (20.9 %)
Ischemic stroke/TIA	53 (18.1 %)	176 (23.5 %)	126 (30.3 %)	79 (45.4 %)	171 (21.1 %)	173 (30.1 %)	39 (29.8 %)	51 (44.3 %)

**Table 3:** Associations between risk factors and WMH degrees

*N* = 1632	PVH				SDWMH			
	OR (95 % CI)				OR (95 % CI)			
	Degree 1 vs 0	Degree 2 vs 0	Degree 3 vs 0	*P*	Degree 1 vs 0	Degree 2 vs 0	Degree 3 vs 0	*P*
Age^a^	2.22 (1.88–2.62)	3.65 (3.01–4.43)	7.75 (5.93–10.13)	< 0.001	1.81 (1.61–2.05)	2.45 (1.97–3.05)	3.83 (2.96–4.95)	< 0.001
Male gender	0.79 (0.57–1.09)	1.06 (0.73–1.53)	1.51 (0.94–2.41)	0.004	0.95 (0.75–1.22)	0.82 (0.55–1.22)	0.77 (0.50–1.17)	0.56
Hypertension	1.96 (1.39–2.76)	2.56 (1.68–3.92)	4.06 (2.12–7.80)	< 0.001	1.88 (1.37–2.58)	2.65 (1.38–5.08)	2.27 (1.16–4.43)	< 0.001
eGFR^b^	0.89 (0.78–1.01)	0.92 (0.80–1.06)	0.75 (0.61–0.92)	0.04	0.96 (0.87–1.07)	0.87 (0.72–1.06)	0.93 (0.75–1.14)	0.50
Diabetes mellitus	1.25 (0.91–1.72)	0.98 (0.68–1.40)	0.84 (0.52–1.33)	0.08	1.16 (0.91–1.47)	0.71 (0.46–1.10)	0.83 (0.53–1.31)	0.09
Hypercholesterolemia	1.00 (0.75–1.34)	0.85 (0.61–1.19)	0.76 (0.50–1.17)	0.37	1.05 (0.84–1.32)	1.14 (0.77–1.67)	0.66 (0.43–1.02)	0.16
Smoking	0.83 (0.58–1.20)	0.85 (0.56–1.28)	0.97 (0.58–1.61)	0.70	1.12 (0.85–1.47)	1.03 (0.65–1.65)	0.85 (0.51–1.41)	0.70
Atrial fibrillation	1.00 (0.42–2.39)	1.02 (0.40–2.60)	1.05 (0.36–3.01)	1.00	0.56 (0.31–1.01)	0.67 (0.27–1.69)	1.23 (0.57–2.65)	0.12
Ischemic heart disease	0.69 (0.41–1.17)	1.04 (0.60–1.80)	0.80 (0.41–1.54)	0.15	0.91 (0.63–1.31)	1.68 (1.01–2.79)	1.52 (0.89–2.61)	0.05
Ischemic stroke/TIA	1.09 (0.76–1.56)	1.42 (0.96–2.10)	2.58 (1.62–4.11)	< 0.001	1.36 (1.06–1.76)	1.27 (0.83–1.94)	2.35 (1.53–2.59)	0.001

Models of multinomial logistic regression adjusted on age, gender and hypertension. In each model, PVH or SDWMH was considered as the dependent variable categorized in 4 degrees (0–3), and degree 0 was the reference category

^a^For age, the OR estimates the association related to an increase of 1 SD

^b^For eGFR, the OR estimates the association related to increase of 30 ml/min/1.73 m^2^

The proportion of individuals with hypertension tended to increase with the degree of WMH; Similarly, subjects with hypertension tended to have higher odds of having higher degrees of PVH and SDWMH (*P* < 0.0001 for both PVH and SDWMH) as compared with normotensive subjects (Tables 2 and 3).

We also observed that the median serum creatinine level increased with WMH degree and median GFR decreased while the degree of WMH increased (Table 2). After adjusted on age, gender and hypertension, patients having each 30 ml/min/1.73 m^2^ increase in eGFR was associated with 75 % of risk of having degree 3 of WMH in periventrical areas as compared with having degree 0. However, this inverse association was not found when look at WMH in deep white matter (OR: 0.93, 95 % CI 0.75–1.14; *P* = 0.50, Table 3). There was no obvious change after further adjustment on diabetes mellitus, hypercholesterolemia, history of ischemic heart disease and ischemic stroke/TIA. (Model 2, data not shown).

No significant associations were observed between WMH and gender, smoking status, diabetes, hypercholesterolemia, history of atrial fibrillation. Subjects with history of ischemic heart disease had higher risk of SDWMH (*P* = 0.05, Table 3), especially SDWMH of degree2 versus degree 0 (OR:1.68, 95 % CI 1.01–2.79, Table 3), but after further adjustment for diabetes mellitus, hypercholesterolemia and eGFR, the value of 1.0 was included in the confidence interval (Model 2, data not shown). Subjects with history of ischemic stroke/TIA had more WMH in both periventricular and subcortical area (*P* < 0.001, Table 3), especially the highest degree(For PVH, OR: 2.58, 95 % CI 1.62–4.11; For SDWMH, OR:2.35, 95 % CI 1.53–3.59, Table 3). Further adjustment for diabetes mellitus, hypercholesterolemia and eGFR did not change the ORs obviously.

## Discussion

This study, performed in a large cohort of patients with AIS/TIA, found that renal function, as measured by eGFR, is inversely associated with WMH in periventrical areas. This association was independent of age, hypertension and other vascular risk factors. However, no association was found between eGFR and WMH in subcortical deep white matter.

The association between decreased GFR and increased WMH is in line with a study of 378 patients with ischemic stroke, which reported that eGFR < 60 mL/min/1.73 m^2^ was associated with severe WMLs independent of age and sex [8]. Most studies in the general population also found this similar association. These results suggested that kidney impairment might serve as a predictive marker for the presence of white matter lesions. However, our finding is in contrast with another study of 523 subjects with acute ischemic stroke which reported no significant association between eGFR and WMH volume [7]. The discrepancy may be explained by the varied definitions of clinical characteristics and the different confounders corrected for from study to study. On the other hand, stroke patients may have more risk factors and more complicated pathogenesis underlying WMH burden accumulation than general population, so there are more controversies about the correlation between GFR and WMH in patients with acute ischemic stroke.

We also found in our sample that the severity of both PVH and SDWMH were significantly associated with age, hypertension and a history of ischemic stroke/TIA. This result is consistent with previous studies showing that WMH burden is greater in individuals with common cerebrovascular risk factors [17]. Though these vascular risk factors are also predictors for GFR decline [18], we cannot determine whether the association between declined GFR and WMH observed in our study is common consequence from shared risk factors or represents a causative relationship, considering our research design.

In our study, the severity of WMH was evaluated separately in two areas and those which located in deep white matter was found not relate to renal function. Our findings have suggested distinct involvement of renal function in the process of WMH accumulation in two locations. Previous studies have also reported that different mechanism involved in the development of PVH and SDWMH, and PVHs are more likely due to diminished cerebral vasomotor reactivity and subsequent hypoperfusion, while SDWMHs are related to microangiopathy [12, 19, 20]. However, only a few studies detected the correlation of GFR with PVH and SDWMH separately. A recent study which distinguished the relationship of GFR with PVH and SDWMH in a large cohort of patients without stroke history showed that eGFR < 60 ml/min/1.73 m^2^ is significantly associated with both PVH and SDWMH [21]. In contrast with our study, they targeted population without history of stroke or other cardiovascular events, and the proportion of subjects with potential risk factors in their study is low, thus the contribution of renal dysfunction to WMH may be augmented.

There are several limitations should be noted in our study. First, considered that it was a multicenter designed study and the serum creatinine concentration had been analyzed in different laboratories, there may be bias of measurement. However, the comparability of serum creatinine concentration detected in different lab was widely accepted in previous studies, and all of the involved centers have rigid quality control process of their clinical and biochemical tests, we think this may not change our results. Second, the WMH assessment had been performed using visual rating scale. Although also widely used, visual rating may be less precise or consistent than quantitative evaluation. Last, as we have excluded subjects with severe renal dysfunction, the proportion of patients with eGFR < 60 ml/min/1.73 m^2^ is lower (only 3.6 %) in our cohort than previous reported (28.1 % in one study and 40.2 % in another) [8, 22], which may underestimate the association of eGFR over WMH.

## Conclusion

In conclusion, we demonstrated that kidney function is associated with WMH in patients having acute ischemic stroke or TIA. Furthermore, this association was found only in WMH located around ventricles. Further studies are needed to improve our understanding of this cerebro-renal coexisting impairment.

## Abbreviations

AF: Atrial fibrillation
AIS: Acute ischemic stroke
CKD: Chronic kidney disease
DM: Diabetes mellitus
eGFR: Estimated glomerular filtration rate
IHD: Ischemic heart disease
PVH: Periventricular white matter hyperintensity
SDWMH: Deep subcortical white matter hyperintensity
TIA: Transient ischemic attack
WMH: White matter hyperintensity
WMH: White matter hyperintensity
